# Procyanidin A1 from Peanut Skin Exerts Anti-Aging Effects and Attenuates Senescence via Antioxidative Stress and Autophagy Induction

**DOI:** 10.3390/antiox14030322

**Published:** 2025-03-07

**Authors:** Yajing Li, Lan Xiang, Jianhua Qi

**Affiliations:** College of Pharmaceutical Sciences, Zhejiang University, Yu Hang Tang Road 866, Hangzhou 310058, China; 12019045@zju.edu.cn (Y.L.); lxiang@zju.edu.cn (L.X.)

**Keywords:** aging, peanut skin, procyanidin A1, cell senescence, antioxidative stress, autophagy, PI3K/Akt signaling pathway

## Abstract

The aging population is steadily increasing, with aging and age-related diseases serving as major risk factors for morbidity, mortality, and economic burden. Peanuts, known as the “longevity nut” in China, have been shown to offer various health benefits, with peanut skin extract (PSE) emerging as a key compound of interest. This study investigates the bioactive compound in PSE with anti-aging potential and explores its underlying mechanisms of action. Procyanidin A1 (PC A1) was isolated from PSE, guided by the K6001 yeast replicative lifespan model. PC A1 prolonged the replicative lifespan of yeast and the yeast-like chronological lifespan of PC12 cells. To further confirm its anti-aging effect, cellular senescence, a hallmark of aging, was assessed. In senescent cells induced by etoposide (Etop), PC A1 alleviated senescence by reducing ROS levels, decreasing the percentage of senescent cells, and restoring proliferative capacity. Transcriptomics analysis revealed that PC A1 induced apoptosis, reduced senescence-associated secretory phenotype (SASP) factors, and modulated the phosphatidylinositol 3-kinase (PI3K)/protein kinase B (Akt) signaling pathway. The antioxidative capacity of PC A1 was also evaluated, showing enhanced resistance to oxidative stress in PC12 cells by reducing reactive oxygen species (ROS) and malondialdehyde (MDA) levels and increasing superoxide dismutase (SOD) activity. Moreover, PC A1 induced autophagy, as evidenced by an increase in fluorescence-labeled autophagic compartments and confirmation via Western blot analysis of autophagy-related proteins. In addition, the treatment of an autophagy inhibitor abolished the antioxidative stress and senescence-alleviating effects of PC A1. These findings reveal that PC A1 extended lifespans and alleviated cellular senescence by enhancing oxidative stress resistance and inducing autophagy, positioning it as a promising candidate for further exploration as a geroprotective agent.

## 1. Introduction

Aging is a gradual and irreversible biological process characterized by a progressive loss of physiological integrity, which leads to declining function and increased susceptibility to age-related diseases such as cancer, cardiovascular disorders, and neurodegenerative diseases. With a global population that is living longer and becoming older, age-related diseases have emerged as significant contributors to morbidity, mortality, and social and economic burdens [1]. Despite the inevitability of death, the rate of aging can be modulated, thus making healthy aging attainable. Numerous compounds have demonstrated significant anti-aging activity, particularly natural products, which often exhibit superior safety and efficacy [2]. Therefore, the exploration of anti-aging molecules derived from natural products presents great potential for the prevention and treatment of aging and age-related diseases.

During the screening of active compounds with anti-aging potential, aging models play a crucial role in evaluating the efficacy of potential therapeutics and understanding their interactions with biological systems. The yeast *Saccharomyces cerevisiae* has been a prominent model organism for studying pathways relevant to aging. Two different paradigms of aging have been established using yeast: the replicative lifespan (RLS) and chronological lifespan (CLS). The RLS of a yeast cell, which is based on the replicative potential of the cells, is defined as the number of daughter cells produced from a single mother cell before death. This metric may correlate with the aging of mitotically active cells in multicellular organisms [3]. The K6001 strain, a genetically modified strain of yeast derived from W303, is particularly useful for conducting RLS assays, as only the mother cells can reproduce offspring in glucose medium, whereas daughter cells cannot [4]. The second paradigm, CLS, measures the duration of survival for non-dividing yeast cells and may parallel the aging of non-dividing cells in higher organisms [5]. However, the yeast chronological senescence does not perfectly align with cellular senescence in mammals. To address this, a yeast-like CLS assay for mammalian cells has been developed to evaluate chronological senescence [6]. Significantly, the same signal transduction pathways that drive yeast-like chronological senescence also influence aging in other organisms. Inhibitors that attenuate the senescent phenotype are found to decelerate yeast-like chronological senescence, establishing this model as a valuable tool for drug discovery focused on anti-aging effects [6].

Cell senescence is a hallmark of aging [7]. Senescence cells accumulate in aged organisms and contribute to the progression of aging and age-related diseases. Cell senescence is a state characterized by irreversible cell cycle arrest. In addition to cell cycle blockade and proliferation defects, senescent cells also have the following features: increased cell size and flattening, impaired mitochondrial function and membrane integrity, elevated levels of reactive oxygen species (ROS), heightened activity of senescence-associated β-galactosidase (SA-β-gal) at a pH of 6, the development of a senescence-associated secretory phenotype (SASP), and nuclear alterations such as DNA damage. Numerous stressors can induce cellular senescence, including replicative stress, genotoxic agents, oncogene activation, oxidative stress, and metabolic stress [8]. Cell senescence has attracted increasing attention, and it is considered a potential target for preventing or treating age-related diseases and extending healthspan. Senotherapeutic strategies that target senescent cells can be classified into two categories: senolytic treatments, which involve the clearance of senescent cells, and senomorphic treatments, which aim to reduce the effects of SASP [9].

ROS are generally small, short-lived, and highly reactive molecules formed by the incomplete one-electron reduction in oxygen. ROS include superoxide, peroxide, and hydroxyl radicals and singlet oxygen. When tightly controlled, ROS serve as signaling molecules. However, when the redox balance is disrupted, excess ROS can lead to oxidative stress, resulting in damage to lipids, proteins, DNA, and carbohydrates. Antioxidants are substances that play a crucial role in delaying, preventing, or removing oxidative damage to target molecules. A variety of antioxidants are present in biological systems, including enzymes (such as superoxide dismutase (SOD)) and various small molecules. The supplementation of antioxidants has been advocated as a strategy to reduce cellular oxidative stress and potentially extend lifespans in different organisms [10,11].

Autophagy is a cellular process that delivers cytoplasmic substrates, including proteins, DNA, and organelles, to lysosomes for degradation. This process can be broadly divided into several stages: initiation, nucleation, elongation, fusion, degradation, and recycling. Autophagy promotes cellular growth and development, protects cells from metabolic stress and oxidative damage, and plays a crucial role in maintaining cellular homeostasis, as well as the synthesis, degradation, and recycling of cellular products [12,13]. Research has shown that autophagy capacity declines with aging, and the regulation of autophagy is vital for lifespan maintenance [14]. Several pharmacological autophagy inducers, such as rapamycin (Rapa), spermidine, flavonoid 4,4′-dimethoxychalcone, and urolithin A, have been shown to prolong lifespans [15,16,17,18].

*Arachis hypogaea* Linn., commonly known as peanut or groundnut, is a significant oilseed and food crop in many tropical and subtropical regions. It is extensively processed into oil, snacks, and peanut paste. China stands as the largest producer of peanuts, accounting for approximately 38% of global production [19]. In Chinese culture, peanuts are referred to as the “longevity nut”. The skin of the peanut, a by-product of the roasting process, has been utilized in Traditional Chinese Medicine (TCM) for centuries to treat various disorders, including hemophilia, hemorrhage, primary and secondary thrombocytopenic purpura, ulcers, inflammation, kidney issues, and hypertension. Recent studies have demonstrated that peanut skin extracts (PSEs) exhibit anti-obesity, anti-atherosclerotic, anti-inflammation, and antioxidant effects in mice. Additionally, PSE contributes to the maintenance of gut microbiota [20,21,22,23]. In this study, a compound that is capable of prolonging the yeast RLS was isolated from the skin of *Arachis hypogaea* Linn. This compound was identified as procyanidin A1 (PC A1) through spectral analysis, with comparisons made to published data. The anti-aging effects and underlying mechanisms of PC A1 were further explored, with findings indicating that it alleviates cell senescence, enhances oxidative stress resistance, and induces autophagy.

## 2. Materials and Methods

### 2.1. General

Analytical pure reagents (methanol, ethanol, dichloromethane) were acquired from Sinopharm Chemical Reagent Co., Ltd. (Shanghai, China). Chromatographic-grade methanol was obtained from the TEDIA Company, LLC. (Fairfield, OH, USA). Silica gel (200–300 mesh) was purchased from the Yantai Research Institute of Chemical Industry (Yantai, China). Reversed-phase C18 (Octadecylsilyl, ODS) silica gel (Cosmosil 75C18-OPN) was from Nacalai Tesque, Inc. (Kyoto, Japan). Develosil ODS-UG-5 (Nomura Chemical Co., Ltd., Aichi, Japan) and Supersil Phenyl (Dalian Elite Analytical Instruments Co., Ltd., Dalian, China) packed columns were used for the isolation and purification of the natural products. Thin-layer chromatography (TLC) analysis was performed using the TLC silica gel plates (Yantai Jiangyou Silicone Development Co., Ltd., Yantai, China) and TLC silica gel 60 RP-18 F254s 25 glass plates (0.25 mm) (Merck KGaA, Darmstadt, Germany). CD_3_OD (Cambridge Isotope Laboratories Inc., Andover, WA, USA) was used as the solvent for ^1^H NMR. ^1^H NMR spectra and HR ESI-TOF-MS data were obtained using a Bruker AV III-500 spectrometer (Bruker Corporation, Karlsruhe, Germany) and Agilent 6224A LC/MS (Agilent Technologies Inc., Beijing, China), respectively.

The following reagents and compounds were purchased from the indicated manufacturer and used in biological experiments: dimethyl sulfoxide (DMSO) from Merck KGaA (Darmstadt, Germany). Resveratrol (Res), rapamycin (Rapa), etoposide (Etop), and chloroquine (CQ) were obtained from Shanghai Yuanye Bio-Technology Co., Ltd. (Shanghai, China). SBI-0206965 was from MedChemExpress LLC. (Shanghai, China).

### 2.2. Isolation and Structure Identification of PC A1

The PSE was prepared according to the previous literature [20]. Under the guidance of K6001 yeast replicative lifespan assay, 1 g of PSE was subjected to ODS open-column chromatography with a methanol/water solvent system (10/90, 30/70, 50/50, 70/30, 100/0). Fractions were combined into 11 fractions according to TLC analysis, and the active fraction (348.1 mg) was obtained from a 30% aqueous methanol elution. Then, 100 mg of this fraction was taken out and subjected to silica open-column chromatography with a dichloromethane/methanol elution system (100/0, 90/10, 70/30, 50/50, 30/70, 10/90, 0/100). Then, 7 samples were obtained. Sample 1 (15.4 mg), obtained from dichloromethane/methanol (90/10), was purified first through HPLC (Develosil ODS-UG-5 (ϕ·10 × 250 mm), methanol: water = 18:100, 100 min, 3 mL/min, 280 nm) to obtain sample 2 (5 mg, t_R_ = 75 min). Sample 2 was then purified again by another HPLC (Supersil Phenyl (ϕ·10 × 200 mm), methanol: water = 20:100, 20 min, 3 mL/min, 280 nm) to obtain PC A1 (3 mg, t_R_ = 16 min) as colorless needles. The isolation scheme of PC A1 from PSE is shown in Supplementary Figure S1. The chemical structure of PC A1 was identified by comparing the ^1^H NMR spectra and MS with the literature [24]: ^1^H NMR (500 MHz, CD_3_OD): *δ*_H_ = 7.13 (1H, d, *J* = 2.1 Hz), 7.01 (1H, dd, *J* = 8.3, 2.1 Hz), 6.91 (1H, s), 6.81 (3H, m), 6.08 (1H, s), 6.06 (1H, d, *J* = 2.3 Hz), 5.95 (1H, d, *J* = 2.3 Hz), 4.73 (1H, d, *J* = 7.8 Hz), 4.23 (1H, d, *J* = 3.5 Hz), 4.15 (1H, m), 4.07 (1H, d, *J* = 3.5 Hz), 2.94 (1H, dd, *J* = 16.5, 5.6 Hz), and 2.57 (1H, dd, *J* = 16.4, 8.3 Hz). The ^1^ H NMR spectrum of PC A1 is shown in Supplementary Figure S2. High-resolution ESI-TOF-MS *m/z* 577.1344 was calculated for C_30_H_25_O_12_ [M+H]^+^ 577.1341. The high-resolution ESI-MS chromatograms of PC A1 is shown in Supplementary Figure S3.

### 2.3. Cell Lines

The PC12 cell line was purchased from the Cell Bank of the Chinese Academy of Sciences (Shanghai, China) and cultured in Dulbecco’s Modified Eagle Medium (DMEM, Cellmax Cell Technology (Beijing) Co., Ltd. (Beijing, China)) supplemented with 10% horse serum (Gibco (Herndon, VA, USA)), 7.5% fetal bovine serum (Cellmax Cell Technology (Beijing) Co., Ltd. (Beijing, China)), and 1% antibiotic–antimycotic solution (Beijing Solarbio Science & Technology Co., Ltd. (Beijing, China)). The NIH/3T3 cell line was generously given from MeilunBio (Dalian, China) and cultured in DMEM containing 10% fetal bovine serum and 1% antibiotic–antimycotic solution. All cells were cultured at 37 °C in a humidified incubator of 5% CO_2_. Etoposide-induced senescence (ETIS) was triggered in NIH/3T3 and PC12 cells after culturing for 2 d in the presence of different concentrations of Etop (0.3 and 7.5 µM, respectively). Oxidative stress was triggered in PC12 cells after culturing for 2 h in the presence of 0.7 mM H_2_O_2_. All cell lines tested negative for microbial contamination and were routinely authenticated with STR assays.

### 2.4. Yeast Replicative Lifespan Assay

K6001 yeast was cultured in galactose liquid medium, which comprised 3% galactose, 2% hipolypeptone, and 1% yeast extract. Following incubation for 24 h with continuous shaking (180 rpm, 28 °C), a total of 4000 cells were washed and then evenly distributed on yeast peptone dextrose (YPD) agar plates, which were formulated with 2% glucose, 2% hipolypeptone, 1% yeast extract, and 2% agar and supplemented with Res or varying concentrations of PC A1. After incubation for 48 h at 28 °C, forty microcolonies from the agar plate were randomly selected for observation under an optical microscope (Olympus, Tokyo, Japan), and the number of daughter cells produced by one mother cell was counted.

### 2.5. Yeast-like Chronological Lifespan Assay

The yeast-like chronological lifespan (CLS) assay was performed according to the reference [6]. The principle of this assay is based on the observation that, over time, a densely overgrown cell culture loses its ability to survive and re-enter the growth phase when transferred to a fresh nutrient-rich medium. This loss of viability occurs in a density-dependent and time-dependent (chronological) manner, reflecting the aging process of the cells. In detail, PC12 cells were seeded at a high density of 8 × 10^4^ cells per well in 96-well plates, with each well containing 0.2 mL of culture medium. After 24 h, the culture medium was replaced with serum-free medium supplemented with either 0.5% DMSO (vehicle control), 200 nM of rapamycin (Rapa, positive control), or procyanidin A1 (PC A1) at concentrations of 1, 3, and 10 µM. After 4 days, the medium, including any floating cells, was carefully aspirated. The adherent cells were then trypsinized using 0.2 mL of trypsin solution. Subsequently, an equivalent volume of the cell culture (4 µL of aliquot), representing approximately 2% of the total adherent (viable) cells, was transferred into 6-well plates containing 4 mL of fresh medium per well. The cells were cultured for an additional 15 days for colony formation. To assess cellular viability, the resulting colonies were fixed with 4% paraformaldehyde for 15 min and subsequently stained with a 0.1% (*w*/*v*) crystal violet solution for 20 min. Following staining, the cells were thoroughly rinsed with water and allowed to air dry. The number of viable colonies, which serves as a quantitative indicator of chronological lifespan, was enumerated and subsequently analyzed for statistical significance.

### 2.6. Cell Viability Assay

An MTT assay was performed to measure the cell viability. In general, 5000 cells were seeded into each well of a 96-well plate and cultured under the indicated treatment. Then, 100 µL of serum-free DMEM containing 500 µg/mL of MTT (Richu BioScience Co., Ltd. (Shanghai, China)) was added and incubated for 4 h followed by the removal of the medium carefully and an addition of 100 µL of DMSO. The plates were read with the absorbance at 570 nm using a plate reader (BioTek Synergy H1, Agilent, Winooski, VT, USA).

### 2.7. Senescence-Associated β-Galactosidase (SA-β-gal) Assay

SA-β-gal staining was performed using the senescence β-galactosidase staining kit (Beyotime Biotechnology Inc., Shanghai, China), adhering strictly to the manufacturer’s protocols. For the assessment of SA-β-gal activity, approximately 50,000 PC12 cells or 20,000 NIH/3T3 cells were seeded into each well of a 24-well plate. Then, cells were exposed to various test samples; 0.5% dimethyl sulfoxide (DMSO) was employed as the negative control, while Rapa served as the positive control. After 24 h, cells were treated with Etop for 2 days. After the treatment, cells were gently washed, fixed, and stained with the provided dyeing solution as directed. Cells were examined under a bright-field microscope (BX63, Olympus, Japan, 20× objective). SA-β-gal^+^ percentages were quantified by counting the number of SA-β-gal-positive cells per visual field.

### 2.8. Cell Proliferation Assay

The 5-Ethynyl-2′-deoxyuridine (EdU) incorporation assay was conducted to evaluate cellular proliferation. For this purpose, the BeyoClick™ EdU Cell Proliferation Kit with Alexa Fluor 488, supplied by Beyotime Biotechnology Inc. in Shanghai, China, was utilized, and the assay was carried out in accordance with the manufacturer’s guidelines. Following treatment, the cells were incubated with EdU at a concentration of 10 µM for 2 h and subsequently fixed and permeabilized, then cells were treated with the click reaction additive solution for 30 min. Following this step, the cells were counterstained with Hoechst 33342 for 10 min. After a thorough washing process, the EdU-positive cells were visualized using a fluorescence microscope (BX63, Olympus, Japan) equipped with a 20× objective lens. The proliferating cells were then quantified based on the fluorescence signals observed.

### 2.9. RNA Extract, RNA-Seq, and Data Analysis

NIH/3T3 cells (4 × 10^5^ cells) at passage 5 were seeded into each 10 cm dish. The next day, the cells were treated with or without 10 µM of PC A1 for 2 days before exposure to 0.3 µM of Etop for 2 days. The control group was treated with the same medium containing an equal amount of DMSO. Total RNA was extracted using the TRIzon reagent (Jiangsu Cowin Biotech Co., Ltd. (Taizhou, China)).

The processes of quality control, cDNA library construction, and sequencing were expertly handled by ShenZhen BGI Genomics Co., Ltd. (Shenzhen, China). Three biological replicates were used for each group. Quality control of the RNA samples was conducted using a Fragment Analyzer, while sequencing was performed on the DNBSEQ platform. The raw data obtained from sequencing were filtered using SOAPnuke to obtain clean data. The clean reads were then aligned to the reference genome using HISAT and to the gene set using Bowtie2. The reference species used for this study was *Mus musculus,* and the specific reference genome version is GCF_000001635.27_GRCm39, sourced from the NCBI database. Differential gene expression analysis was conducted using the DESeq2 method, identifying genes with a Q value (adjusted *p* value) ≤ 0.05 and a log_2_(Fold Change (FC)) ≥ 0.5 or ≤−0.5 as differentially expressed genes (DEGs). For comprehensive data analysis and visualization, including the Kyoto Encyclopedia of Genes and Genomes (KEGG) pathway enrichment analysis and the Gene Ontology (GO) biological process enrichment analysis, the online bioinformatic platform tool Dr. Tom provided by BGI (https://biosys.bgi.com (accessed on 18 November 2024)) was utilized. Additionally, volcano plots and heatmaps were carried out using another online platform, CNSKnowall (https://cnsknowall.com (accessed on 20 February 2025)).

### 2.10. ROS Assay

A ROS assay was performed using a ROS Assay Kit (Beyotime Biotechnology Inc., Shanghai, China), following the manufacturer’s instructions. Generally, 50,000 cells were seeded into each well of a 24-well plate. Then, cells were treated with PC A1 (1, 3, 10, and 30 µM) for 18 h, and then with H_2_O_2_ for 2 h or Etop for 2 days. Then, DCFH-DA, at a final concentration of 10 µM, was added and incubated with cells for 30 min. After washing with phosphate-buffered saline (PBS), fluorescence was observed using a fluorescence microscope (IX53, Olympus, Japan, 20× objective).

### 2.11. MDA Quantifications and SOD Assay

Approximately 10^6^ of the PC12 cells were seeded in a 60 mm culture dish. Then, cells were treated with Res (10 µM) or PC A1 (1, 3, 10, and 30 µM) for 18 h, and then with 0.7 mM H_2_O_2_ for another 2 h. The MDA quantification and SOD assay were determined using the MDA assay kit and SOD assay kit (Nanjing Jiancheng Bioengineering Institute, Nanjing, China) in accordance with the manufacturer’s instructions, respectively.

### 2.12. Autophagy Detection

At first, approximately 80,000 PC12 cells were seeded in each well of a 24-well plate. After 24 h, cells were treated with CQ (10 µM, as a negative control), Rapa (500 nM) plus CQ (as a positive control), and PC A1 (at doses of 0, 3, 10, and 30 µM) plus CQ. After 18 h, the cells were stained with the CYTO-ID^®^ Autophagy Detection Kit (Enzo Life Sciences, Inc., New York, NY, USA), according to the manufacturer’s instructions. Briefly, the culture medium was removed, and the cells were rinsed twice with an assay buffer supplemented with 5% fetal bovine serum. Subsequently, 250 µL of assay buffer, containing 0.2% green detection reagent and 0.1% Hoechst 33342 nuclear stain, was added to each well. Following a 30 min incubation, the cells were fixed, washed with the assay buffer, and then examined under a confocal microscope (BX61, Olympus, Japan) with a 20× objective lens.

### 2.13. Western Blot Analysis

Whole-cell lysates were prepared using the RIPA lysis buffer containing a 1% complete protease inhibitor cocktail (Jiangsu Cowin Biotech Co., Ltd. (Taizhou, China)), 1% phosphatase inhibitor cocktail II, and 1% phosphatase inhibitor cocktail III (Abcam Limited. (Cambridge, UK)) and homogenized and centrifuged at 12,000× *g* for 20 min at 4 °C. The protein concentration of cell lysates was determined by the BCA protein assay kit. Cell lysates were added with thr SDS-PAGE sample loading buffer and heated for 10 min at 100 °C. Then, 20 µg of protein of each sample was separated by electrophoresis on sodium dodecyl sulfate (SDS) polyacrylamide gels and transferred to an Immun-Blot PVDF membrane (Bio-Rad Laboratories Inc., Hercules, CA, USA). Membranes were incubated with primary and then secondary antibodies. Primary antibodies against phosphoinositide 3-kinase (PI3K) (#4249, 1:1000), phospho-PI3K (#4228, 1:1000), mammalian target of rapamycin (mTOR, #2983, 1:1000), phospho-mTOR (#5536, 1:1000), unc-51 like autophagy activating kinase 1 (ULK1, #8054, 1:1000), phospho-ULK1 (Ser757, #14202, 1:1000), p62 (#5114, 1:1000), microtubule-associated protein 1 light chain 3 (LC3B, #2775, 1:1000), protein kinase B (Akt, #9272, 1:1000), and phospho-Akt (#9271, 1:1000) were procured from Cell Signaling Technology, Inc. (Boston, MA, USA). Beclin-1 (#HA721216, 1:1000) was purchased from Hangzhou HuaAn Biotechnology Co., Ltd. (Hangzhou, China). p21 (#AP021, 1:200) was from Beyotime Biotechnology Co., Ltd. (Shanghai, China). β-Actin (#CW0096, 1:1000) was from Jiangsu Cowin Biotech Co., Ltd. (Taizhou, China). HRP-conjugated goat anti-rabbit IgG (#CW0103S, 1:5000) and anti-mouse IgG (#CW0102S, 1:5000) were from Jiangsu Cowin Biotech Co., Ltd. (Taizhou, China). Finally, antigens were visualized using the SuperPico ECL Chemiluminescence Kit (Nanjing Vazyme Biotech Co., Ltd., Nanjing, China). The Bio-Rad ChemiDoc^TM^ MP Imaging System (Bio-Rad Laboratories, Inc., Hercules, CA, USA) was used for the detection of proteins of interest, and the blot density was quantified utilizing Image Lab software (Version 6.1, Bio-Rad Laboratories, Inc., Hercules, CA, USA).

### 2.14. Statistical Analysis

Statistical analyses were performed using the GraphPad Prism software (Version 9.0, GraphPad Software, LLC, San Diego, CA, USA). To assess differences among multiple groups, ordinary one-way ANOVA was conducted, followed by Dunnett’s multiple comparisons test for post hoc analysis. For pairwise comparisons between the treatment group and the control group in the replicative lifespan assay, two-tailed, unpaired Student’s *t*-tests were employed. Statistical significance was defined as *p* < 0.05. Each experiment was repeated three times, and data for each experiment are shown as mean ± SEM.

## 3. Results

### 3.1. PC A1 Extended the Replicative Lifespan of K6001 Yeast and Yeast-like Chronological Lifespan of PC12 Cells

**Figure 1:**
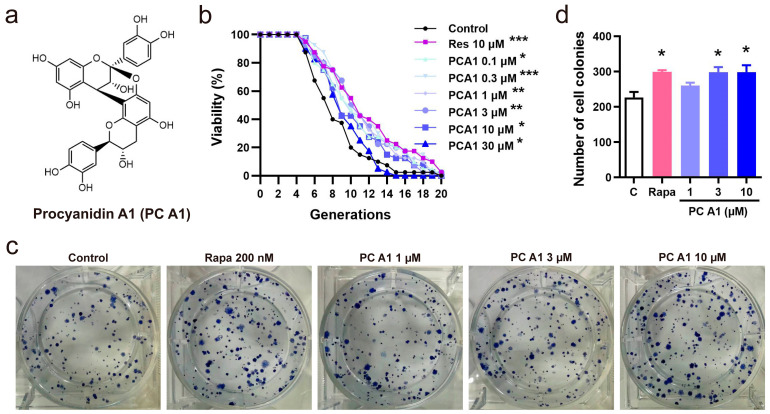
Procyanidin A1 (PC A1) isolated and purified from peanut skin extended the replicative lifespan of K6001 yeast and the yeast-like chronological lifespan of PC12 cells. (**a**) The chemical structure of PC A1. (**b**) The effect of PC A1 on the replicative lifespan of K6001 yeast. Resveratrol (Res) at 10 µM was used as the positive control. The experiment was conducted three independent times, with each replicate involving 40 individual mother cells per treatment group. The replicative lifespan was determined by counting the number of daughter cells produced by each mother cell. To assess the significance of the results, a two-sided Student’s *t*-test was used to compare the number of daughter cells in each treatment group to the control group. (**c**,**d**) The colony formation of PC12 cells treated with PC A1 (1, 3, and 10 µM) and rapamycin (Rapa, 200 nM) for testing the yeast-like chronological lifespan of PC12 cells. The representative images of colony-forming units (CFUs) (**c**) and quantification (**d**) are shown. Rapamycin (Rapa) was used as the positive control. Experiments were repeated three times. Data represent mean ± SEM. Significant difference was obtained with ordinary one-way ANOVA followed by Dunnett’s multiple comparisons test. *, **, and *** indicate significant differences at *p* < 0.05, *p* < 0.01, and *p* < 0.001 compared with the control (C) group, respectively.

Due to its convenience and speed, the K6001 yeast RLS assay was utilized as a guide to isolate compounds with potential anti-aging activity. In this study, PC A1 was isolated and purified from PSE under the guidance of the yeast RLS system. The isolation scheme and the chemistry structure of PC A1 are presented in Supplementary Figure S1 and Figure 1a, respectively. In the yeast RLS assay, Res was employed as a positive control for its stimulating effects on SIRTI and its ability to prolong the lifespan in yeast [25]. The results in Figure 1b demonstrate that PC A1 (0.1, 0.3, 1, 3, and 10 µM) and Res significantly slowed the replicative aging of yeast. However, PC A1 at 30 µM exhibited toxicity to the yeast. Furthermore, yeast-like CLS assays were conducted on PC12 cells to evaluate the lifespan-extending effect of PC A1 at the mammalian cell level. The results in Figure 1c,d indicate that PC A1 (1 and 3 µM) and Rapa (as a positive control) increased the number of colonies, demonstrating that PC A1 could prevent the senescence-mediated decline of clonogenic survival in highly confluent PC12 cells [6]. Collectively, these findings suggest that PC A1 prolongs both yeast RLS and yeast-like CLS in mammalian cells.

### 3.2. PC A1 Alleviated Cellular Senescence Induced by Etoposide

#### 3.2.1. PC A1 Alleviated Cellular Senescence Induced by Etoposide in PC12 Cells

**Figure 2:**
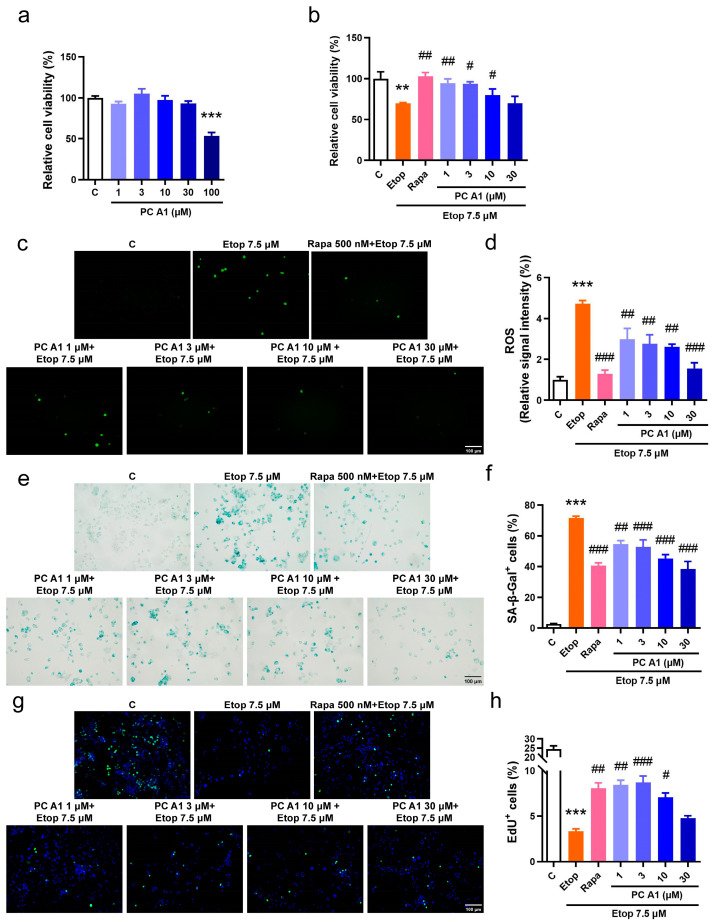
Procyanidin A1 (PC A1) alleviated Etop-induced senescence in PC12 cells. (**a**) The cell viability of PC12 cells after treatment with PC A1 (1, 3, 10, 30, and 100 µM) for 24 h. (**b**–**h**) PC12 cells were pre-treated with PC A1 (1, 3, 10, and 30 µM) for 24 h followed by 7.5 µM of etoposide (Etop) for 48 h. The cellular viability was determined (**b**), and ROS level (**c**,**d**), senescence-associated β-galactosidase (SA-β-gal) staining (**e**,**f**), and cell proliferation (**g**,**h**) are pictured and quantified. Proliferating cells were labeled with green fluorescence, and nuclei were stained with Hoechst 33342. Scale bar: 100 µm. Data represent mean ± SEM, *n* = 3 for each group. ** and *** indicate significant differences at *p* < 0.01 and *p* < 0.001 compared with the control (C) group, respectively; #, ##, and ### indicate significant differences at *p* < 0.05, *p* < 0.01, and *p* < 0.001 compared with the etoposide (Etop) group, respectively.

The evaluation of cellular senescence as a hallmark of aging was conducted. Cellular senescence is characterized by elevated levels of ROS, an increased expression of SA-β-gal, and arrested cell proliferation. Etoposide (Etop), a chemotherapy agent, inhibits topoisomerase II, destroys the rejoining of DNA after superhelical unwinding, induces DNA damage, and subsequently leads to cell cycle arrest and senescence. Therefore, Etop was employed to model the pathological conditions associated with cellular senescence. The PC12 cell line, derived from a pheochromocytoma of the rat adrenal medulla, is widely utilized in neuroscience research, including studies on neuroprotection, neurosecretion, neuroinflammation, and synaptogenesis [26]. Recent studies also demonstrated that PC12 cells are valuable in simulating the cellular changes associated with nervous system aging [27]. Initially, the viability of PC12 cells following treatment with PC A1 was assessed. The results indicated that PC A1 (at concentrations of 1, 3, 10, and 30 µM) did not exhibit any significant cytotoxic effects on normal PC12 cells, whereas the treatment of PC A1 at 100 µM resulted in a 50% reduction in cellular viability (Figure 2a). Subsequently, PC12 cells were pre-treated with PC A1 (at 1, 3, 10, and 30 µM) for one day and then exposed to Etop (7.5 µM) for 2 days to evaluate the protective effect of PC A1 against cell senescence. Rapa at 500 nM was used as a positive control due to its known geroprotective effects [15]. Cell viability was assessed using the MTT assay, and the results demonstrated that PC A1 (at 1, 3, and 10 µM) and Rapa (500 nM) significantly mitigated the loss of cellular viability caused by Etop (Figure 2b). Furthermore, the level of ROS was found to be elevated by Etop; however, treatment with PC A1 (1, 3, 10, and 30 µM) and Rapa (500 nM) significantly reduced ROS levels (Figure 2c,d). Subsequently, SA-β-gal staining was performed, as shown in Figure 2e,f, and quantitative analysis revealed that over 70% of Etop-treated cells exhibited a senescent phenotype characterized by SA-β-gal-positive cells. In contrast, the pre-treatment of PC A1 (at 1, 3, 10, and 30 µM) significantly decreased the number of SA-β-gal-positive PC12 cells. Cell cycle arrest, another hallmark of cell senescence, was assessed using the EdU cell proliferation kit. EdU, a thymidine analog, was incorporated into DNA during synthesis, followed by a click reaction with a green fluorescence-labeled azide probe, thereby labeling newly synthesized DNA for the detection of proliferating cells. To visualize all cells, nuclei were stained with Hoechst 33342. As shown in Figure 2g,h, nearly 25% of the PC12 cells in the control group were EdU positive, exhibiting green fluorescence, whereas this percentage dropped to 4% in Etop-induced senescent cells. The pre-treatment of PC A1 (1, 3, and 10 µM) and Rapa (500 nM) protected against the proliferative impairment caused by Etop. These experimental findings suggest that PC A1 effectively prevented cellular viability loss, reduced ROS levels, decreased the number of SA-β-gal-positive senescent cells, and restored proliferative capacity, thereby alleviating Etop-induced senescence in PC12 cells.

#### 3.2.2. PC A1 Alleviated Cell Senescence Induced by Etoposide in NIH/3T3 Cells

**Figure 3:**
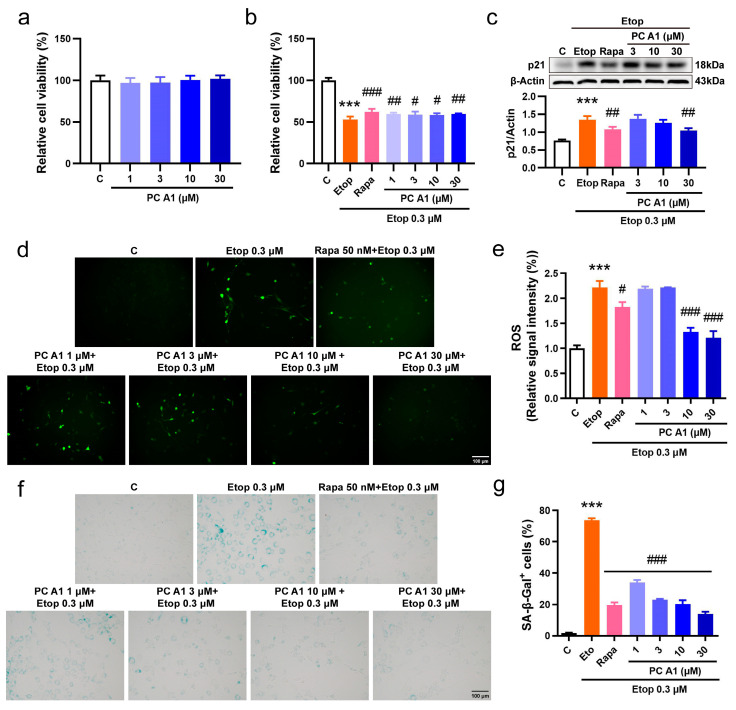
Procyanidin A1 (PC A1) alleviated Etop-induced senescence in NIH/3T3 cells. (**a**) The cell viability of NIH/3T3 cells after treatment with PC A1 (1, 3, 10, and 30 µM) for 24 h. (**b**–**g**) NIH/3T3 cells were pre-treated with PC A1 (1, 3, 10, and 30 µM) for 24 h followed by 0.3 µM of etoposide (Etop) for 48 h. The cellular viability was determined (**b**), the expression levels of p21 were evaluated (**c**), and ROS levels (**d**,**e**) and senescence-associated β-galactosidase (SA-β-gal) staining (**f**,**g**) were pictured and quantified. Scale bar: 100 µm. The experimental samples and controls used for the comparative analysis in (**c**) were run on the same blot/gel. Data represent mean ±SEM, *n* = 3. *** indicates significant differences at *p* < 0.001 compared with the control (C) group; #, ##, and ### indicate significant differences at *p* < 0.05, *p* < 0.01, and *p* < 0.001, compared with the etoposide (Etop) group, respectively.

To further confirm the cell senescence alleviation effect of PC A1, we then induced cell senescence using Etop in the NIH/3T3 cell line. The NIH/3T3 mouse embryonic fibroblast cell line serves as a vital model for in vitro research, particularly in the fields of cellular senescence and cell cycle studies. Considering that NIH/3T3 cells exhibit greater sensitivity to Rapa and Etop, the concentration of Rapa was adjusted to 50 nM to serve as a positive control, whereas the dose of Etop was reduced to 0.3 µM. The effect of PC A1 on cellular viability was also assessed. As shown in Figure 3a, PC A1 (at 1, 3, 10, and 30 µM) demonstrated no toxicity to NIH/3T3 cells. Following this, NIH/3T3 cells were pre-treated with PC A1, subsequently inducing cell senescence with Etop for 2 days, and then we evaluated cellular viability, the expression of p21, ROS levels, and SA-β-gal activity. The results in Figure 3b show that the pre-treatment of PC A1 (1, 3, 10, and 30 µM) and Rapa (50 nM) significantly protected NIH/3T3 cells from the loss of viability. The expression levels of p21, a biomarker of cellular senescence, were then assessed. As shown in Figure 3c, PC A1 at 30 µM and Rapa reduced p21 levels elevated by Etop. The results of ROS assays (Figure 3d,e) and SA-β-gal staining (Figure 3f,g) were consistent with those obtained from the PC12 cells, demonstrating that both PC A1 and Rapa significantly decreased ROS levels and the number of senescent cells, as indicated by SA-β-gal-positive cells. In conclusion, PC A1 alleviated cellular senescence induced by Etop in both PC12 and NIH/3T3 cells.

#### 3.2.3. The PI3K/Akt Signaling Pathway Played a Role in the Alleviation Effect of PC A1 on Cell Senescence

**Figure 4:**
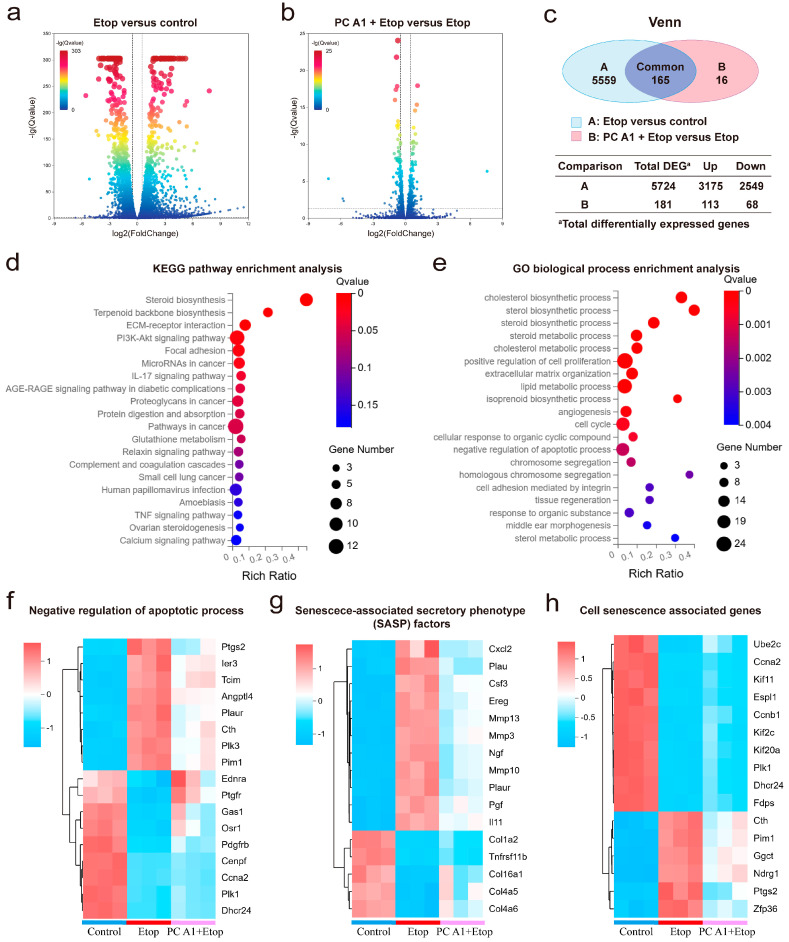
RNA sequencing analysis on senescent NIH/3T3 cells treated with procyanidin A1 (PC A1). NIH/3T3 cells were pre-treated with PC A1 (10 µM) for 24 h followed by 0.3 µM of etoposide (Etop) for 48 h. RNA was extracted from cells lysis for RNA sequencing analysis. (**a**) Volcano plot of gene expression in the Etop group compared to the control group. (**b**) Volcano plot of gene expression in the PC A1 + Etop group compared to the Etop group. In the volcano plot, the two vertical dashed lines indicate log2(FoldChange) values of −0.5 and 0.5, while the horizontal dashed line represents a Q-value threshold of 0.05. Genes with log2(FoldChange) ≥ 0.5 or ≤−0.5 and a Q value ≤ 0.05 were identified as differentially expressed. (**c**) Venn diagram showing the number of differentially expressed genes (DEGs, with log_2_FoldChange (FC) ≥ 0.5 or ≤−0.5, Q value ≤ 0.05, calculated by raw count value) and overlapped genes between A and B (A. Etop group versus control group; B. PC A1 + Etop group versus Etop group). (**d**) KEGG pathway enrichment analysis of the overlapped 165 common differentially expressed genes. (**e**) GO biological processes associated with the 165 common differentially expressed genes. (**f**) Heatmaps of genes associated with negative regulation of the apoptosis process. (**g**) Heatmaps of genes associated with senescence associated secretory phenotype (SASP) factors in different groups. (**h**) Heatmaps of cell senescence-related genes according to the CellAge database. *n* = 3 for each group. *n* stands for the number of samples in a group.

To further understand how PC A1 mitigates cell senescence, gene expression profiling of NIH/3T3 cells was performed through RNA sequencing. As shown in Figure 4a–c, a total of 5724 differentially expressed genes (DEGs) (log_2_FoldChange (FC) ≥ 0.5 or ≤−0.5, Q value ≤ 0.05) were identified between the Etop-treated group and control group, with 3175 genes upregulated and 2549 genes downregulated in Etop-treated cells compared to the control group. Additionally, 181 DEGs were found between the Etop + PC A1 group and the Etop-only group, with 113 genes upregulated and 68 downregulated in the PC A1 pre-treatment group compared to the Etop group.

The 165 genes that were differentially expressed in both comparisons were analyzed using the KEGG pathways and GO enrichment analysis. As shown in Figure 4d, the KEGG pathway analysis identified several significantly enriched pathways associated with the effects of PC A1 treatment on Etop-induced senescence. Among these pathways, the PI3K/Akt signaling pathway, focal adhesion, ECM–receptor interaction, and pathways in cancer were prominently represented. Notably, the PI3K/Akt signaling pathway exhibited the highest number of enriched genes and a low Q value. Therefore, we propose that the PI3K/Akt signaling pathway plays a crucial role in the anti-aging effect of PC A1.

The DEGs were annotated using the GO terms from the GO database to enhance our understanding of their molecular characteristics. As shown in Figure 4e, the DEGs were annotated using the GO terms from the GO database to further discover their molecular characterization. The DEGs were categorized into three main areas: biological processes, molecular functions, and cellular components. Within the biological process category, the DEGs were implicated in the positive regulation of cell proliferation, the cell cycle, and the negative regulation of the apoptotic process. We further analyzed the transcriptomic expression profile. As shown in Figure 4f, 17 genes were associated with the negative regulation of the apoptotic process. We observed that the expression of eight genes associated with apoptosis resistance increased with Etop treatment but was reduced upon treatment with PC A1. These genes included *Ptgs2*, *Ier3*, *Tcim*, *Angpt14*, *Plaur*, *Cth*, *Plk3,* and *Pim*. Additionally, we noted a significant upregulation of several SASP factors (including *Cxcl2*, *Plau*, *Csf3*, *Ereg*, *Mmp3*, *Mmp10*, *Mmp13*, *Ngf*, *Plaur*, *Pgf*, and *Il11*) during cellular senescence, which were substantially downregulated by PC A1. Four genes (*Col16a1*, *Col1a2*, *Col4a5*, and *Col4a6*), encoding members of the collagen family, along with *Tnfrsf11b,* were found to be downregulated in senescent cells but substantially upregulated by treatment with PC A1 (Figure 4g). Furthermore, a search of gene expression databases, specifically CellAge [28], revealed 16 genes associated with cell senescence that were regulated by Etop but alleviated by PC A1 treatment (Figure 4h). Overall, these findings confirm that PC A1 ameliorates cell senescence induced by Etop by promoting apoptosis in senescent cells, regulating the gene transcription levels of SASP factors and restoring proliferative capacity. Moreover, the PI3K/Akt signaling pathway appears to play a role in the protective effect of PC A1 against cellular senescence.

### 3.3. PC A1 Remedied Oxidative Stress in H_2_O_2_-Exposed PC12 Cells

**Figure 5:**
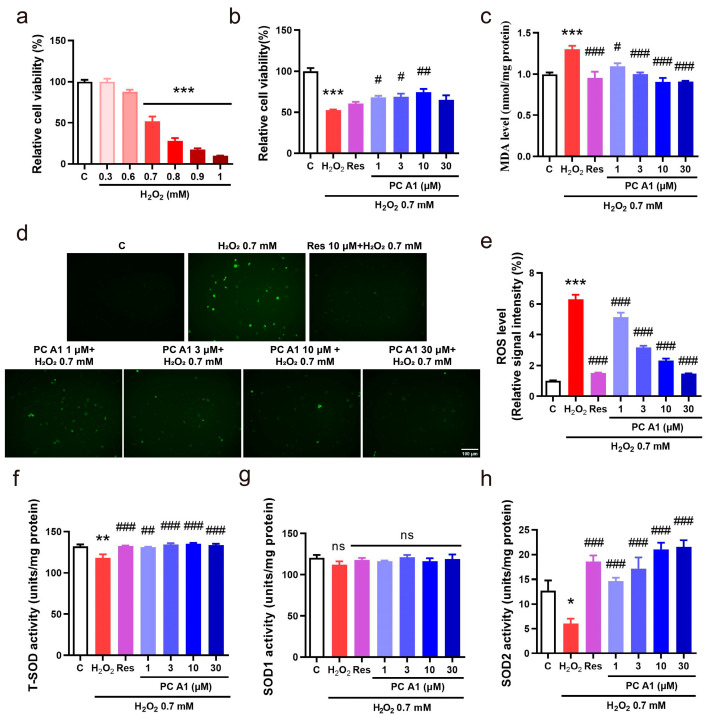
Antioxidative stress effect of procyanidin A1 (PC A1). (**a**) PC12 cell viability change caused by different doses of H_2_O_2_. (**b**–**h**) PC A1 reduced H_2_O_2_-induced toxicity and attenuated oxidative stress. PC12 cells were pre-treated with PC A1 (1, 3, 10, and 30 µM) for 24 h followed by 0.7 mM H_2_O_2_ for 2 h. Then, the cellular viability (**b**), MDA level (**c**), ROS level (**d**), and its digital result (**e**) and total SOD (**f**), SOD1 (**g**), and SOD2 activities (**h**) of PC12 cells were assessed. Scale bar: 100 µm. Data represent mean ± SEM, *n* = 3 for each group. ns, *, **, and *** indicate significant differences at *p* > 0.05, *p* < 0.05, *p* < 0.01, and *p* < 0.001 compared with the control (C) group, respectively; ns, #, ##, and ### indicate significant differences at *p* > 0.05, *p* < 0.05, *p* < 0.01, and *p* < 0.001 compared with the H_2_O_2_ group, respectively.

In organisms, the aging process is associated with progressive mitochondrial dysfunction, leading to an increased production of ROS. The pathological level of ROS contributes to further mitochondrial deterioration and widespread cellular damage, including harm to DNA, proteins, and lipids, which are significant factors in aging and age-related diseases [29,30]. Therefore, we investigated the ability of PC A1 to reduce ROS and protect PC12 cells from oxidative stress. H_2_O_2_, the most commonly used endogenous source of cellular oxidative stress, was employed to induce this condition. Initially, the appropriate concentration of H_2_O_2_ was pre-tested. After incubating cells with varying doses of H_2_O_2_ for 2 h, cell viability was assessed using the MTT assay. As shown in Figure 5a, H_2_O_2_ at 0.7 mM resulted in a 50% reduction in cell viability compared to the control and was chosen as an appropriate concentration for further experiments. Cells were subsequently treated with different doses of PC A1 for 24 h, followed by exposure to 0.7 mM H_2_O_2_ for 2 h. The findings in Figure 5b indicate that the reduction in cell viability caused by H_2_O_2_ was alleviated by the pre-treatment with PC A1 (1, 3, and 10 µM). Res was utilized as a positive control due to its well-established antioxidant properties [31]. The level of MDA, a common indicator in oxidative stress studies, was also assessed. As shown in Figure 5c, pre-treatment with Res (10 µM) and PC A1 (1, 3, 10, and 30 µM) significantly lowered the elevated MDA levels induced by H_2_O_2_. Furthermore, ROS levels were evaluated using DCFH-DA, a probe that undergoes hydrolysis and oxidation to produce fluorescent DCF. As shown in Figure 5d,e, H_2_O_2_ produced a marked increase in ROS levels, which were significantly reduced by pre-treatment with Res and PC A1 (at 1, 3, 10, and 30 µM). SOD is a crucial endogenous antioxidant enzyme that scavenges superoxide anion free radicals and protects cells from oxidative damage. The activities of total SOD, SOD1, and SOD2 were measured. The results in Figure 5f–h demonstrate the PC A1 enhanced SOD2 activity but did not affect SOD1 activity. In conclusion, PC A1 protects PC12 cells from oxidative damage induced by H_2_O_2_ by increasing SOD2 activity and reducing both ROS and MDA levels.

### 3.4. PC A1 Induced Autophagy in PC12 Cells

**Figure 6:**
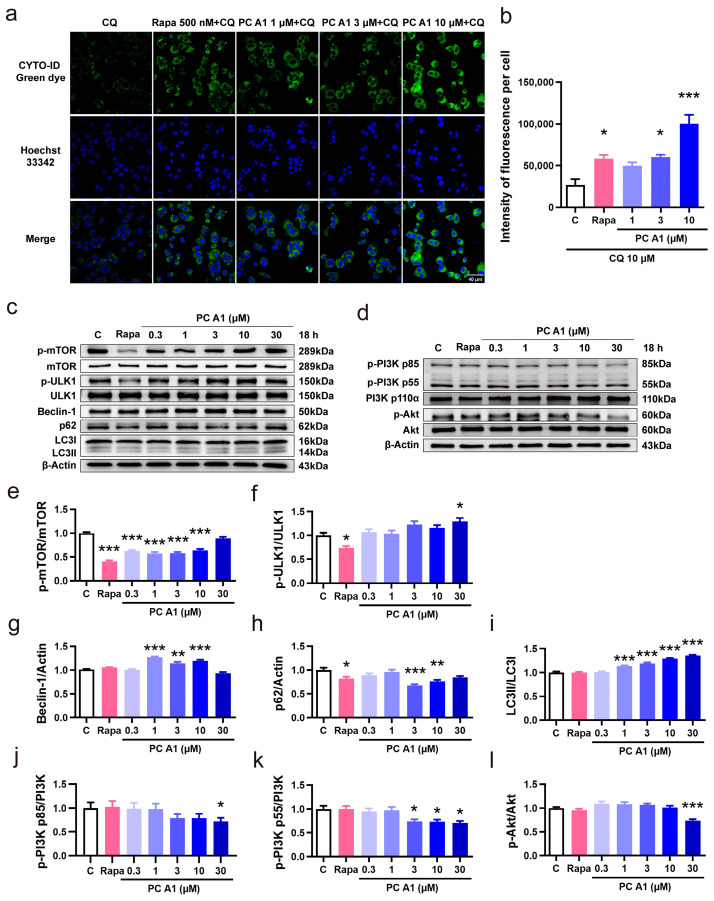
Procyanidin A1 (PC A1) induced autophagy in PC12 cells. (**a**) Staining of the autophagic vesicles with a fluorescent dye in PC12 cells after treatment with rapamycin (Rapa) and PC A1. Scale bar: 40 µm. (**b**) The fluorescence intensity quantification results of (**a**). (**c**) The Western blot results of p-mTOR (Ser2448), mTOR, p-ULK1 (Ser757), ULK1, Beclin-1, p62, and LC3 compared with β-Actin in PC12 cells after treatment with 500 nM of Rapa and different doses of PC A1 for 18 h. (**d**) The Western blot results of p-PI3K (p85 (Tyr458)/p55 (Tyr199)), PI3K, p-Akt (Ser473), and Akt in PC12 cells after treatment with 500 nM of Rapa and different doses of PC A1 for 18 h. (**e**–**i**) The digital Western blot results of p-mTOR (Ser2448)/mTOR (**e**), p-ULK1 (Ser757)/ULK1 (**f**), Beclin-1 (**g**), p62 (**h**), and LC3II/I (**i**). (**j**–**l**) The digital Western blot results of p-PI3K p85 (Tyr458)/PI3K (**j**), p-PI3K p55 (Tyr199)/PI3K (**k**), and p-Akt (Ser473)/Akt (**l**). The samples used for the Western blot analysis in (**c**,**d**) on different proteins are derived from the same experiment or parallel experiments and the blots are processed in parallel. *, **, and *** represent significant differences compared with the negative control (*p* < 0.05, *p* < 0.01, and *p* < 0.001, respectively). The experiments were repeated three times, and data from each experiment are displayed as mean ± SEM.

Impaired microautophagy has been proposed as a hallmark of aging [7]. Autophagy is the cellular process by which portions of the cell, including macromolecules and entire organelles, are degraded within lysosome. CYTO-ID^®^ green dye, a fluorescent probe, serves as an effective marker for vesicles produced during autophagy. To assess the capacity of PC A1 to induce autophagy, CQ was employed to block the conversion from autophagosomes to autolysosomes by elevating the lysosomal/vacuolar pH. Rapa was used as a positive control for its known capacity to induce autophagy by inhibiting TOR (in particular, TOR complex 1) activity [15]. As shown in Figure 6a,b, Rapa (500 nM) and PC A1 (3 and 10 µM) significantly increased the fluorescent intensity of autophagic vacuoles. Given that the conversion from autophagosomes to autolysosomes was blocked by CQ, we conclude that PC A1 effectively induces autophagy in PC12 cells.

Then, the expressions of autophagy-associated proteins mTOR, ULK1, Beclin-1, p62, and LC3 were evaluated through Western blot analysis to confirm the intracellular autophagy. PC12 cells were incubated with varying doses of PC A1 (0.3, 1, 3, 10, and 30 µM) for 18 h to assess dose dependence. As illustrated in Figure 6c, the levels of phospho-mTOR (Ser2448) were reduced by both PC A1 and Rapa. Meanwhile, the levels of Beclin-1 and the ratio of LC3II/LC3I were upregulated, whereas the p62 levels were decreased in response to PC A1. The digital results of Figure 6c are shown in Figure 6e–i. Additionally, PC12 cells were incubated with 3 µM of PC A1 for varying time periods to assess time dependence, and the Western blot results for mTOR, ULK1, Beclin-1, p62, and LC3 are shown in Supplementary Figure S4a. These results further confirm that PC A1 induces autophagy in a dose- and time-dependent manner.

Then, the PI3K/Akt signaling pathway, serving as an upstream signaling pathway in the induction of autophagy, was also explored. As shown in Figure 6d, PC A1 decreased the level of the phosphorylated form of PI3K p85 (Tyr458) and p55 (Tyr199), as well as Akt (Ser473), in a dose-dependent manner. The digital results are presented in Figure 6j–l. Time dependence was similarly assessed, and the Western blot results are shown in Supplementary Figure S4b. These findings suggest that PC A1 enhances autophagy, in which the PI3K/Akt signaling pathway was involved.

### 3.5. Autophagy Inhibitor Abolished Antioxidative Stress and Cell Senescence Alleviation Effects of PC A1

**Figure 7:**
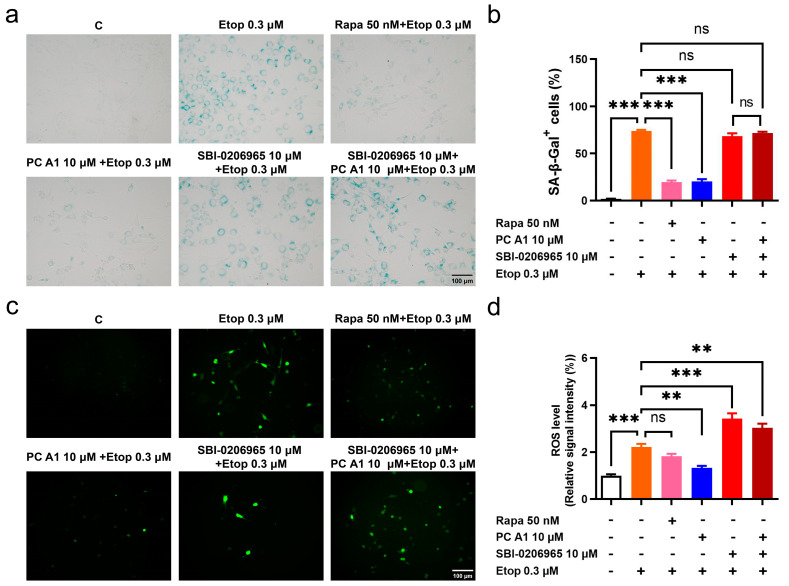
The autophagy inhibitor SBI-0206965 abolished antioxidative stress and the cell senescence alleviation effect of procyanidin A1 (PC A1). (**a**–**d**) NIH-3T3 cells were pre-treated with PC A1 at 10 µM with or without SBI-0206965 for 24 h followed by 0.3 µM of etoposide (Etop) for 48 h. Rapa (50 nM) was used as a positive control. The senescence-associated β-galactosidase (SA-β-gal) staining (**a**,**b**) and ROS level (**c**,**d**) are pictured and quantified. Scale bar: 100 µm. Data represent mean ± SEM, *n* = 3 for each group. ns, **, and *** indicate significant differences at *p* > 0.05, *p* < 0.01, and *p* < 0.001 compared with the designated group, respectively.

Studies have indicated that many features of aging are interconnected. The impairment of autophagy or genetic defects in autophagy in young cells can lead to a loss of proteostasis, heightened mitochondrial dysfunction, and increased oxidative stress, ultimately resulting in cellular senescence [12]. To test whether the antioxidative stress and cell senescence alleviation effects of PC A1 could be negated by impaired autophagy, SA-β-gal staining and ROS assays were conducted. The selective ULK1 kinase inhibitor SBI-0206965, used to inhibit autophagy in vitro, was administered [32]. The results in Figure 7a indicated that PC A1 at 10 µM distinctly reduced the SA-β-gal^+^ cell percentage promoted by Etop. However, SBI-0206965 negated this effect of PC A1. The evaluation of ROS yielded results consistent with the SA-β-gal assay. As shown in Figure 7b, Etop at 0.3 µM induced elevated levels of ROS in NIH/3T3 cells; pre-treatment with Rapa (50 nM) and PC A1 (10 µM) reduced ROS levels. Conversely, SBI-0206965 abrogated the ROS scavenging effect of PC A1. The results confirm that there is a crosstalk between autophagy and oxidative stress, as well as cellular senescence. The inhibition of autophagy can obstruct the ROS scavenging and cell senescence elimination effects of PC A1.

## 4. Discussion

The aging population is steadily increasing, highlighting the importance of identifying phytochemical senotherapeutics compounds with significant potential. In China, peanuts are known as the “longevity nut” and PSE is recognized as a form of TCM. Modern pharmacological studies have demonstrated that PSE offers multiple benefits against various age-related vulnerabilities. However, the potential anti-aging effects of the bioactive molecules from PSE remain not fully understood. In this study, we utilized K6001 yeast RLS as a guiding model to isolate PC A1 from PSE. Our research findings indicate that PC A1 can extend the RLS, as well as the CLS of mammalian cells. Additionally, we observed that PC A1 provides a protective effect against cellular senescence and oxidative stress and induces autophagy.

PC A1 is classified as an A-type procyanidin dimer, which is a part of the proanthocyanidin class of flavonoids. The constituent units of procyanidins are catechin and/or epicatechin. A-type procyanidins (linked via C_4_-C_6_ or C_4_-C_8_ and C_2_-O-C_7_) and B-type procyanidins (linked via C_4_-C_6_ or C_4_-C_8_) are categorized based on the specific interflavan linkages among their constituent units. Previous studies have identified peanut skin as a rich source of procyanidins, including monomers, dimers, trimers, and tetramers, with a particular abundance of A-type procyanidins [33]. Studies have shown that A-type procyanidins demonstrate prebiotic-like, antioxidative, anti-inflammation, anti-diabetic, antiviral, neuroprotective, and autophagy- and apoptosis-inducing effects [34]. Notably, some studies have also indicated that procyanidins may have the potential to extend lifespans and alleviate age-related pathologies [35,36]. However, different procyanidins may exhibit inverse phenotypes. For example, procyanidin C1 was shown to increase ROS in senescent cells, but not for procyanidin B2 [36]. Thereby, the anti-aging mechanism of PC A1 was further investigated.

A fundamental aging mechanism that has attracted increasing attention is cellular senescence. Senescent cells accumulate with age, and if persistent, can adversely affect tissue function due to the SASP that they develop. Despite the cytotoxic microenvironment they create, senescent cells evade death by regulating pro-survival and anti-apoptotic pathways, such as the PI3K/Akt signaling pathway. Senolytic agents, which selectively eliminate senescent cells, and senomorphic agents, which reduce the SASP, have shown promise as interventions for aging and treating age-related diseases [9]. In this study, Etop, a DNA topoisomerase inhibitor, was utilized to induce cellular senescence. This treatment elevated the expression of p21, increased ROS production, resulted in a rise in the number of SA-β-gal-positive cells, and caused cell cycle arrest, while the treatment of PC A1 alleviated these changes (Figure 2 and Figure 3). To further investigate the mechanisms of PC A1, we conducted RNA-sequencing analysis. The results revealed that PC A1 regulates the PI3K/Akt signaling pathway. Furthermore, PC A1 downregulated the expression of anti-apoptosis genes that had been increased by Etop (Figure 4f) and reduced many SASP factors produced by senescent cells (Figure 4g). Thus, it is believed that PC A1 exhibited both senolytic and senomorphic functions, with the PI3K/Akt signaling pathway playing a significant role in the senotherapeutic effects of PC A1.

Oxidative stress is a prevalent theme among the key features associated with the aging process. It can lead to various hallmarks of aging, including the accumulation of damaged proteins, telomere attrition, epigenetic alterations, cellular senescence, and mitochondrial dysfunction, contributing to aging and age-related diseases. While antioxidants may act as scavengers of ROS to maintain the biological redox homeostasis, they may play a protective role in aging and age-related diseases [29]. In this study, H_2_O_2_ was used to induce oxidative stress in PC12 cells, and the treatment of PC A1 significantly improved cell viability under oxidative stress and reduced ROS levels. MDA, a harmful end product of lipid peroxidation, was also assessed as an indicator of oxidative damage. The results indicated that PC A1 decreased MDA production. As a primary defense against ROS-mediated damage, SODs were also evaluated. We observed an increase in total SOD and SOD2 activity following the treatment with PC A1; however, SOD1 activity remained unchanged (Figure 5). Notably, elevated ROS levels were also observed in senescent cells, and pre-treatment with PC A1 significantly reduced ROS levels in both senescent PC12 cells and NIH/3T3 cells (Figure 2c and Figure 3d).

Disabled macroautophagy is one of the hallmarks of aging [7]. The term ‘autophagic flux’ refers to the entire process of autophagic degradation, including the formation of autophagosome and the subsequent degradation of the cargo within lysosomes. To assess the ability to induce autophagy, we blocked the fusion of lysosome and autophagosome using CQ and evaluated the autophagy flux by labeling autophagic compartments with a CYTO-ID fluorescence dye [37]. The results indicated that PC A1 increased autophagy (Figure 6a,b). Consistently, the expression levels of autophagy-related proteins, including p62, Beclin-1, and LC3II/I, also confirmed the activation of autophagy (Figure 6c). Furthermore, the upstream signaling pathway that regulates autophagy was tested through Western blot analysis, which revealed that PC A1 downregulated the levels of phosphorylated PI3K and Akt (Figure 6d).

Studies have demonstrated that impaired autophagy leads to oxidative stress and cellular senescence [12]. In this study, we observed that Rapa, as an autophagy inducer, also reduced ROS levels in senescent cells (Figure 2c and Figure 3d). Conversely, SBI-0206965, a highly selective ULK1 kinase inhibitor and autophagy inhibitor, exacerbated the senescence phenotype. Cells treated with both SBI-0206965 and Etop were even bigger and flatter than those treated with Etop alone (Figure 7a). Additionally, SBI-0206965 elevated ROS levels in senescent cells. Notably, the ability of PC A1 to scavenge ROS and decrease the number of SA-β-gal-positive cells was diminished by SBI-0206965 (Figure 7). These findings support the notion that autophagy deficiency plays a critical role in oxidative stress and cellular senescence. Meanwhile, ULK1, a conserved kinase involved in autophagy initiation, emerges as a necessary component in the anti-aging mechanism of PC A1.

The PI3K/Akt signaling pathway regulates signal transduction and biological processes such as cell growth, proliferation, survival, apoptosis, autophagy, and metabolism [38,39]. Previous research has shown that PI3K inhibition can alleviate aging in Drosophila and reduce cardiac aging and immune senescence in older adults [40,41,42]. In this work, the levels of phosphorylated PI3K and Akt were reduced by PC A1 (Figure 3d). Furthermore, RNA-seq analysis indicated that the PI3K/Akt signaling pathway is implicated in the senescence-alleviating effects of PC A1 (Figure 6d). Therefore, we propose that the PI3K/Akt signaling pathway plays a role in the anti-aging effects of PC A1. However, further data are needed to substantiate this in our future work. Additionally, this investigation into the anti-aging effects of PC A1 is currently limited to the cellular level and requires validation at the animal level. Given the low concentration of PC A1 in peanut skin, alternative sources must be explored for its isolation.

## 5. Conclusions

Overall, our findings highlight the potential of PC A1 as an anti-aging agent. Isolated from PSE, PC A1 demonstrates significant efficacy, including an extension of the RLS of yeast and the CLS of mammalian cells, as well as alleviating cell senescence, mitigating oxidative stress, and inducing autophagy. Furthermore, the PI3K/Akt signaling pathway plays a role in the anti-aging effects of PC A1. Given these promising results, further investigation into PC A1 as a potential geroprotective agent is warranted.

## Data Availability

All figures and data used to support this study are included within this article; further inquiries can be directed to the corresponding authors.

## Supplementary Materials

The following supporting information can be downloaded at: https://www.mdpi.com/article/10.3390/antiox14030322/s1, Figure S1: Isolation scheme of procyanidin A1 (PC A1) from peanut skin extract (PSE). Figure S2: The ^1^H NMR spectrum of procyanidin A1 (PC A1) (500 MHz, CD_3_OD). Figure S3: The HR ESI-TOF-MS chromatogram of procyanidin A1 (PC A1). Figure S4: Procyanidin A1 (PC A1) induced autophagy in PC12 cells, time dependently. (**a**) The Western blot results of p-mTOR (Ser2448), mTOR, p-ULK1 (Ser757), ULK1, Beclin-1, p62, and LC3 compared with β-Actin in PC12 cells after treatment with 500 nM of rapamycin (Rapa) and 3 µM of PC A1 for different times (0, 0.5, 1, 2, 4, 8, 18, 24, and 48 h). (**b**) The Western blot results of p-PI3K (p85 (Tyr458)/p55 (Tyr199)), PI3K, p-Akt (Ser473), and Akt in PC12 cells after treatment with 500 nM of rapamycin (Rapa) and 3 µM of PC A1 for different times (0, 0.5, 1, 2, 4, 8, 18, 24, and 48 h). (**c**–**g**) The digital Western blot results of p-mTOR (Ser2448)/mTOR (**c**), p-ULK1 (Ser757)/ULK1 (**d**), Beclin-1 (**e**), p62 (**f**), and LC3II/I (**g**). (**h**–**j**) The digital Western blot results of p-PI3K p85 (Tyr458)/PI3K (**h**), p-PI3K p55 (Tyr199)/PI3K (**i**), and p-Akt (Ser473)/Akt (**j**). The samples used for the Western blot analysis in (**a**,**b**) on different proteins are derived from the same experiment or parallel experiments and the blots were processed in parallel. *, **, and *** represent significant difference compared with the negative control (*p* < 0.05, *p* <0.01, and *p* < 0.001). The experiments were repeated three times, and data from each experiment are displayed as mean ± SEM.

## Abbreviations

The following abbreviations are used in this manuscript:

CLS	chronological lifespan
CQ	chloroquine
DEGs	differentially expressed genes
EdU	5-ethynyl-2′ -deoxyuridine
Etop	etoposide
ETIS	etoposide-induced senescence
FC	fold change
MDA	malondialdehyde
PC A1	procyanidin A1
PSE	peanut skin extracts
Rapa	rapamycin
Res	resveratrol
RLS	replicative lifespan
ROS	reactive oxygen species
SA-β-gal	senescence-associated β-galactosidase
SASP	senescence-associated secretory phenotype
SOD	superoxide dismutase
TCM	Traditional Chinese Medicine
YPD	yeast peptone dextrose
