# Supplementary material for: Procyanidin A1 from Peanut Skin Exerts Anti-Aging Effects and Attenuates Senescence via Antioxidative Stress and Autophagy Induction
Source: Antioxidants (Basel). 2025 Mar 7;14(3):322. doi: 10.3390/antiox14030322 (PMC11939485; doi:10.3390/antiox14030322)
Supplement: Supplementary file 1 [file antioxidants-14-00322-s001.zip › antioxidants-3509841-supplementary.pdf]

Supplementary Materials

Procyanidin A1 from Peanut Skin Exerts Anti-aging Effects and Attenuates Senescence via Anti-oxidative Stress and Autophagy Induction

Yajing Li [1], Lan Xiang [1], Jianhua Qi [1,*]

[1] College of Pharmaceutical Sciences, Zhejiang University, Yu Hang Tang Road 866, Hangzhou 310058, China; 12019045@zju.edu.cn (Y.L.), lxiang@zju.edu.cn (L.X.)

* Correspondence: qijianhua@zju.edu.cn (J.Q.)

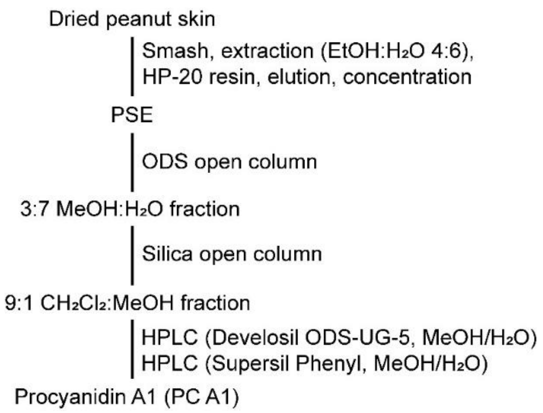

Figure S1. Isolation scheme of procyanidin A1 (PC A1) from peanut skin extract (PSE).

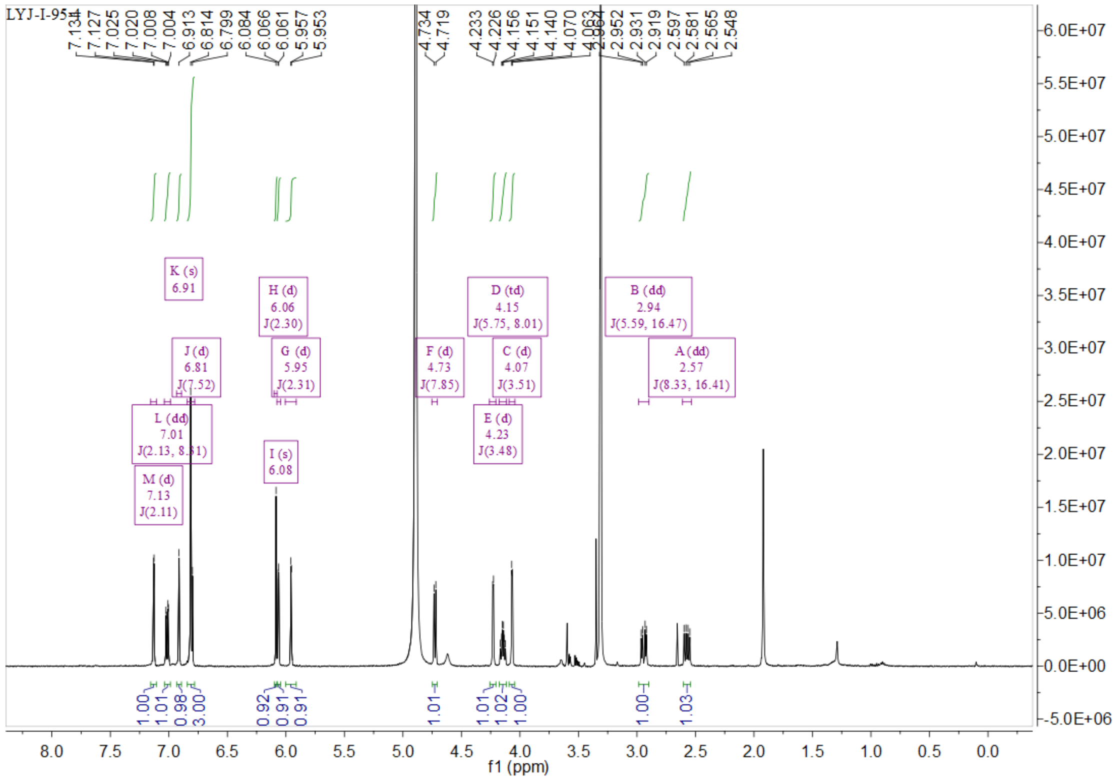

Figure S2. The ¹H NMR spectrum of procyanidin A1 (PC A1) (500 MHz, CD_3OD).

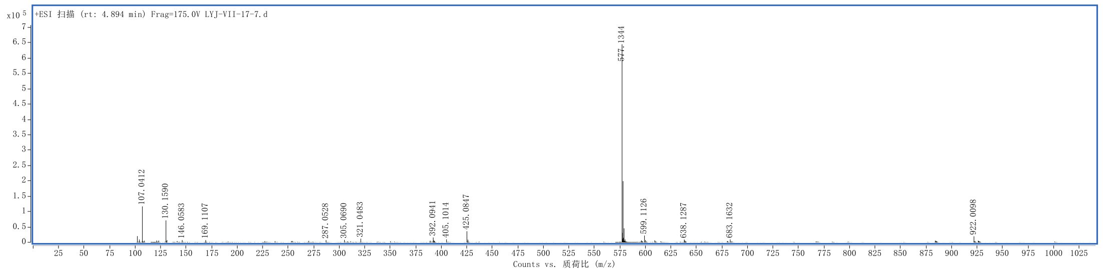

Figure S3. The HR ESI-TOF-MS chromatogram of procyanidin A1 (PC A1).

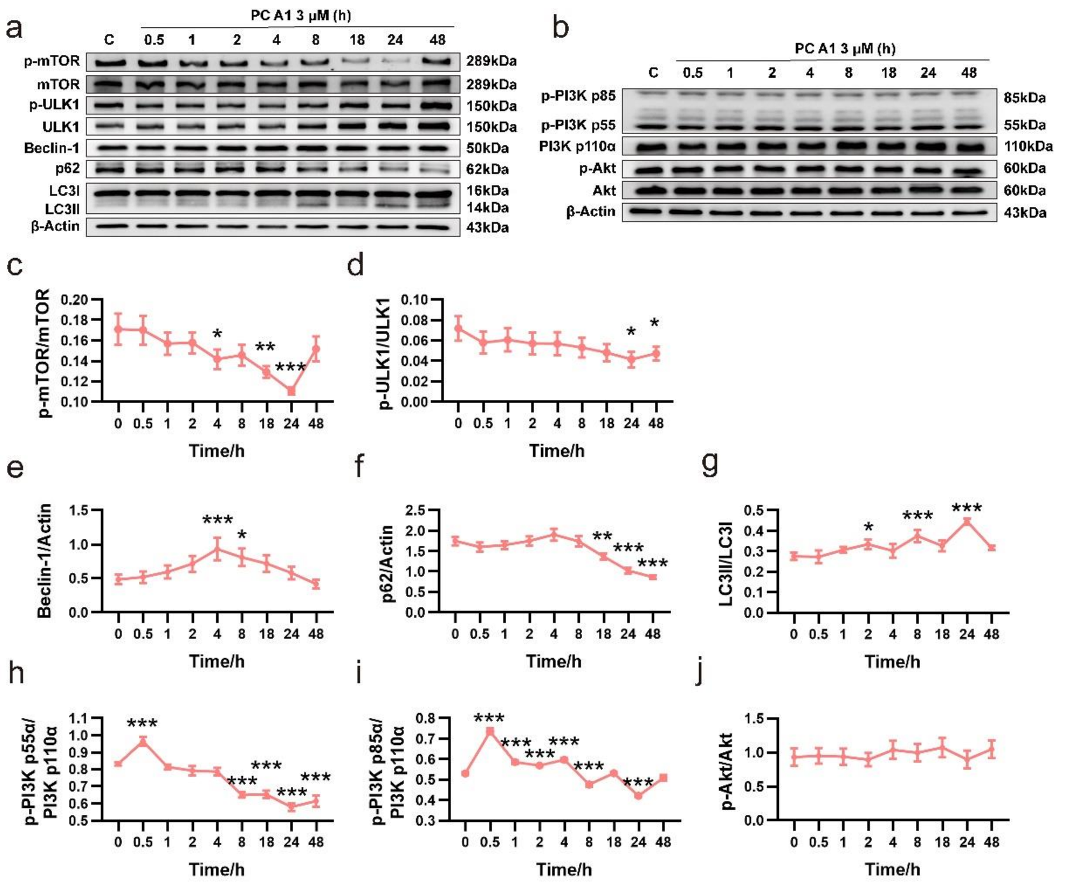

Figure S4. Procyanidin A1 (PC A1) induced autophagy in PC12 cells, time dependently. (**a**) The Western blot results of p-mTOR (Ser2448), mTOR, p-ULK1 (Ser757), ULK1, Beclin-1, p62, LC3 compared with β-Actin in PC12 cells after treatment with 500 nM rapamycin (Rapa) and 3 μM PC A1 for different time (0, 0.5, 1, 2, 4, 8, 18, 24, 48 h). (**b**) The Western blot results of p-PI3K (p85 (Tyr458)/p55 (Tyr199)), PI3K, p-Akt (Ser473), Akt in PC12 cells after treatment with 500 nM rapamycin (Rapa) and 3 μM PC A1 for different time (0, 0.5, 1, 2, 4, 8, 18, 24, 48 h). (**c**–**g**) The digital Western blot results of p-mTOR (Ser2448)/mTOR (**c**), p-ULK1 (Ser757)/ULK1 (**d**), Beclin-1 (**e**), p62 (**f**), LC3II/I (**g**). (**h**–**j**) The digital Western blot results of p-PI3K p85 (Tyr458)/PI3K (**h**), p-PI3K p55 (Tyr199)/PI3K (**i**), p-Akt (Ser473)/Akt (**j**). The samples used for the Western blot analysis in (**a**) and (**b**) on different proteins are derived from the same experiment or parallel experiments and the blots were processed in parallel. *, **, *** represent significant difference compared with negative control ($p < 0.05$, $p<0.01$, $p < 0.001$). The experiments were repeated three times and data from each experiment was displayed as mean ± SEM.
